# At-home laser treatment of oral neuronal disorders: Case reports

**DOI:** 10.4317/jced.53373

**Published:** 2017-04-01

**Authors:** Elisabetta Merigo, Jean-Paul Rocca, Aldo Oppici, Luigi Cella, Carlo Fornaini

**Affiliations:** 1 Micoralis Laboratory EA7354 – University of Nice “Sophia Antipolis”, 24 Avenue des Diables Bleus, Nice - France; 2 “Guglielmo da Saliceto” Hospital, via Taverna, 1o – 29100 - Piacenza – Italy

## Abstract

The neuronal disorders occurring in the oral district are mainly anaesthesia, paraesthesia, hypoesthesia and hyperaesthesia and they may occur frequently after surgical procedures. Medical treatment depends on degree of severity of the nerve injury but, in every case, it must be immediately carried out to reduce immune inflammatory reaction. The aim of this report is to investigate the effectiveness in the recovery of the peripheral nerve lesions of a new laser device recently proposed by the commerce that, due to its reduced size and to be a class I laser according the ANSI classification, may be used at home by the patient himself. Three different cases were treated with this “at-home approach”: complete resolution of symptomatology was obtained after laser treatment with a good compliance for the patient and without reporting any side effect.

** Key words:**Laser, biomodulation, low level laser therapy, oral neuronal disorders, at-home treatment, paresthesia.

## Introduction

The neuronal disorders occurring in the oral district are mainly four: anaesthesia, paraesthesia, hypoesthesia and hyperaesthesia (1): a brief description of each of them is given in the  Table 1.

**Table 1:**
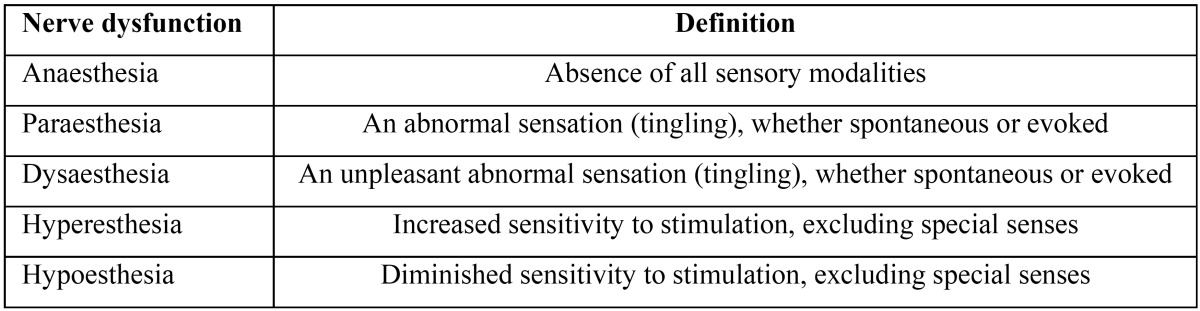
Main neuronal disorders occurring in the oral district are manly four.

The nerve-related complications following oral and dental surgery are being reported with increasing frequency and typically affect the inferior alveolar nerve (IAN; 64.4% of all cases) (2).

Nerve damage can occur during third molar, orthognathic, pre-prosthetic, implant and salivary gland surgery, or during the resection of benign or malignant neoplasms. Endodontic treatment and local anaesthetic injections can also result in nerve damage (3), which can reduce quality of life by affecting speech, chewing and social interaction (4).

The 96% of inferior alveolar nerve (IAN) injuries recover within 4 - 8 weeks after surgery and the recovery rates are apparently independent by gender and only slightly dependent by age (5). Some injuries may be permanent with a duration longer than 6 months, and with symptoms variables from mild hypoesthesia to complete anaesthesia and neuropathic responses resulting in chronic pain (6). One recent study reported that over 50% of lingual or inferior alveolar nerves damaged during surgery had completely healed after 6 months follow-up (7) but, by another different study (8) if the nerve has not healed within 12 months the damage is usually permanent.

Medical treatment depends on degree of severity of the nerve injury but, in every case, it must be immediately carried out to reduce immune inflammatory reaction. In case of mild degree of nerve injury, it may be prescribed a large dose of non-steroidal anti-inflammatory drug (such as 400-600 mg ibuprofen) three times daily for 1 week. In case of moderate or severe nerve injury, the appropriate treatment is a course of oral steroids such as oral dexamethasone 4 mg, two tablets AM for 3 days and one tablet AM for next 3 days or oral prednisolone 1 mg per kg per day (maximum 80 mg). An alternative or additional therapy would be a large dose of non-steroidal anti-inflammatory drug (such as 800 mg ibuprofen) three times daily for 3 weeks. Prescription of these drugs must be undertaken with consideration to the patient’s medical history and caution.

In all cases, additionally diuretics (torasemidum, 10 mg per day, for 5 days), vasodilators (pentoxifylline, 1200 mg per day for 10 days), and B-group vitamins (neurorubine forte lactab once per day for 2 weeks) and antihistaminic drugs (loratadinum 10 mg per day) may be prescribed (9).

In case of clinical improvement, course of nerve recovery drugs were repeated during 3 months period (B-group vitamins, vasodilators) while in some unresponsive cases additional pharmacologic agents were used, including antidepressants, anticonvulsants, antisympathetic agents, and topical medications. Nerve specialists may prescribe also additional physiologic therapies, such as transcutaneous electric nerve stimulation and acupuncture (10).

Low Level Laser Therapy (LLLT) or photobiomodulation is able to increase cell respiration, adenosine triphosphate (ATP) synt-hesis and phosphocreatine resynthesis, reducing also the acidification by accelerating the oxidation of lactate to pyruvate occurred in the mitochondria. This is the reason why LLLT have been suggested also for the recovery of the peripheral nerve lesions (11).

The mechanism of LLLT seems to consist in the alteration of nerve cell activity, inducing upregulation of several neurotrophic growth factors and extracellular matrix proteins, which support neurite outgrowth (12). The increase of GAP-43 immunoreactivity in the early stages of rat sciatic nerve regeneration after phototherapy may explain this effect (13). Snyder *et al.* showed that phototherapy upregulates calcitonin gene-related peptide mRNA expression in facial motor nuclei after axotomy (14). Phototherapy could optimize rate of regeneration, target innervations and neuronal survival of axotomized neurons by means the alteration of the intensity and temporal pattern of injury-induced CGRP expression (15).

The aim of this preliminary study is to investigate the effectiveness in the recovery of the peripheral nerve lesions of a new laser device recently proposed by the commerce that, due to its reduced size and to be a class I laser according the ANSI classification, may be used at home by the patient himself.

In fact, one of the problems related to the LLLT is represented by the necessity, for the patients, to go to the therapist twice/three times weekly for treatments of some minutes. The appearing in the market of new LLLT appliances, cheaper, smaller and able to be used at home by the patients themselves might represent a solution to this problem, giving the possibility to the patients to receive LLLT treatment also daily avoiding the risk of overpower, due to this device has only a time setting.

## Case Reports

Patients realized their own the « at home treatment » with a laser B-Cure (Israël): this device is a class I laser (173 g weight) emitting in the infrared spectrum (808 nm) with an aiming beam green showing the irradiation area of 4,5 cm2 and an output power of 250 mW, emitted in micro-Pulse with a frequency of 15 kHz, for an energy per minute of 14,4 Joules and a Fluence per minute of 3,2 J/cm2. Treatment was realized daily for a session of 15 minutes (total Fluence 48 J/cm2) for extraoral application at the lower lip, chin and the region of mental foramen.

**Figure 1:**
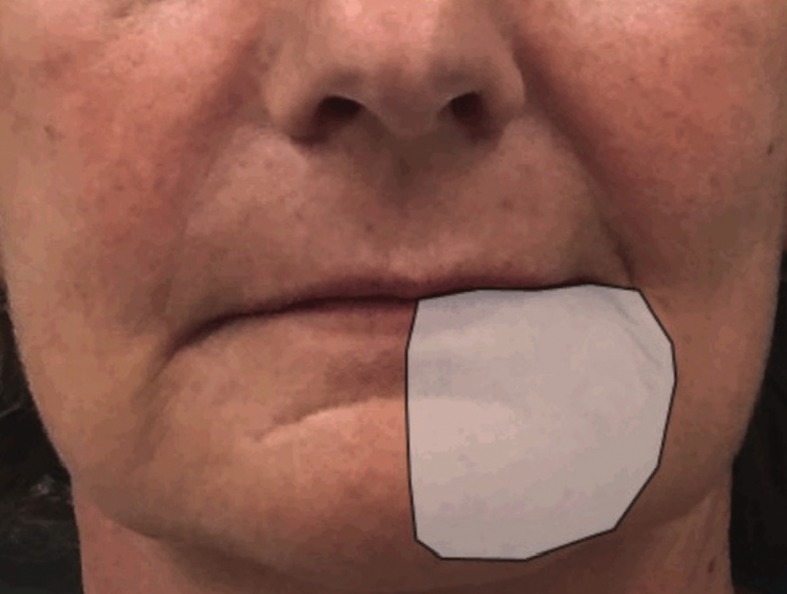
Case 2 patient referred the paraesthesia of the right lower lip until the chin from some months before surgery due to the osteonecrotic disease.

**Figure 2:**
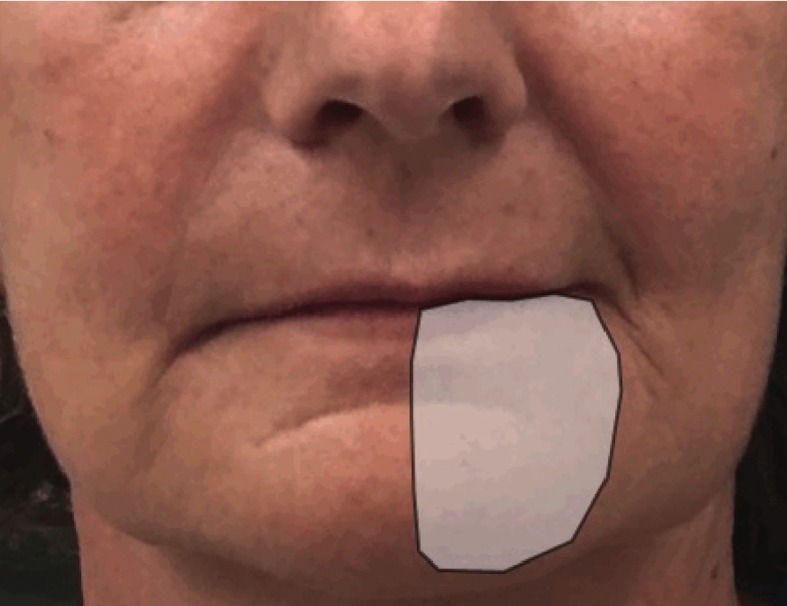
Case 2 patient referred the reduction of the area of paraesthesia after 1 week of LLL treatment.

**Figure 3:**
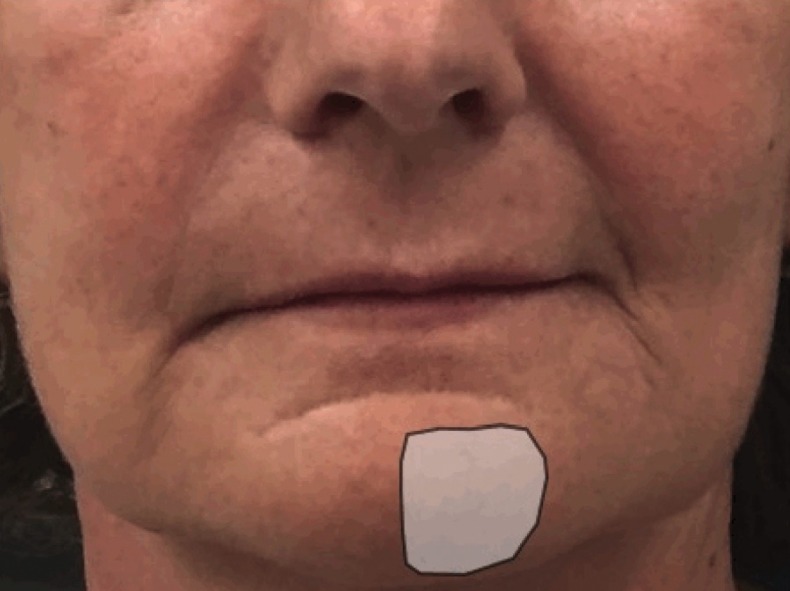
Case 2 patient referred the additional reduction of the area of paraesthesia after 2 weeks of LLL treatment.

The degree of sensory nerve damage was assessed by objective measurements performed by means of a 25 G needle (diameter 0,51 mm) in the injured area. This was then used to map the area of anesthesia and paraesthesia, and the boundary of this region was marked on the skin. A photograph was taken to record the area of damage (Figs. 1-3).

-Case 1

A 55-year-old woman was surgically treated to place in the molar region of her left mandible 2 dental implants: surgery was performed without any intraoperatory complication but the day after surgery she referred the appearance of a paraesthesia of the lower lip in its left side associated in some area to anaesthesia. At the evaluation of the radiographic and TC images the distal part of the dental implant was near but non in contact with lower alveolar nerve.

The day after surgery, the patient started with a daily application of 15 minutes of the laser device B-Cure applied on the cutaneous part of the left mandible.

Just after 3 applications the symptomatology was completely disappeared.

-Case 2

A 55-year-old woman previously surgically treated for a mandibular Osteonecrosis of the Jaws related to bisphosphonates (BRONJ) was routinely evaluated in our Department for the BRONJ follow up: patient referred the paraesthesia of the right lower lip until the chin (Fig. 1) from some months before surgery due to the osteonecrotic disease (Stage 3 on the basis of BRONJ Ruggero classification). Panoramic exam and TC scan, together with the clinical aspect of the oral site, allowed us to exclude a recurrence for BRONJ disease and to formulate the diagnosis of post BRONJ paraesthesia.

The patient, given her informed consent, started with a daily application of 15 minutes of the laser device B-Cure applied on the cutaneous part of the right mandible, lighting for the molar part of the mandible until the chin.

Patient was evaluated every week in order to evaluate the results of the laser treatment: paraesthesia and anaesthesia sensations were localized, by a nociceptive test, in a smaller area after the first (Fig. 2) and second week (Fig. 3) of treatment and completely disappeared after the 3rd week.

-Case 3

A 63-years old man without systemic disease referred to our clinic for the evaluation of his symptomatology described as painful sensation associated to mild paraesthesia of the lower part of the left cheek until the lower lip. Symptomatology was appeared after implant surgery with positioning of an implant at the place of tooth 3.7, it was not improved after the removal of the implant, realized 3 years after implant positioning, and it was not responding to pharmacological treatment.

Laser treatment was performed at-home twice a day for 2 weeks with the complete resolution of symptomatology.

## Discussion

In the literature about the treatment of inferior alveolar nerve injury there is a lack of evidence to support or refute the effects of different interventions and low evidence to support the effects of laser therapy on patient-reported altered sensation.

The results of the presented clinical cases support previous findings supporting the correlation supporting the correlation between LLL treatment and subjective and objective improvement in long-standing neurosensory deficit: the new concept to highlight in our approach, already highlighted for the treatment of other conditions (15), compared with the other ones, is that patient may perform the treatment his own at home, limiting the number of medical control without any problem of protocol parameters and without any side effect. In literature several studies highlighted that microsurgical repair of the nerve injuries can provide moderate to significant clinical neurosensory improvement after surgery, but LLL therapy appears for the previously explained reasons, to be more beneficial and advantageous thanks to the absence of invasivity.
